# Book Reviews

**Published:** 1895-07

**Authors:** 


### Book Reviews.

Suggestive Therapeutics in Psychopathia Sexualis, with Especial Reference to Contrary Sexual Instinct. By Dr. A. von Schrenck-Notzing (Munich, Germany). Authorized translation from the German, by Charles Gilbert Chaddock, M. D., Professor of Diseases of the Nervous System, Marion-Sims College of Medicine, St. Louis; Member of the American Medico-Psychological Association; Attending Neurologist to the Rebekah Hospital, St. Louis, Mo., etc., etc. One volume royal 8vo, 325 pages. Extra cloth, $2.50 net; sheep, $3.50 net. Philadelphia: The F. A. Davis Co., Publishers, 1914-1916 Cherry street. 1895.

This book may be considered very properly as a companion to Krafft-Ebing's recent work, entitled Psychopathia Sexualis. It is true that the views of these two authors are in striking contrast; nevertheless, that is an additional reason why the books should be placed in juxtaposition.

It is more than probable that English medical literature would be quite as well off had neither of these works been translated. The subjects with which they deal are disgusting. It is doubtful, too, whether the class of cases dealt with in this work receive much benefit from treatment. There are underlying factors in their causation that in general thwart therapeutics. Sexual perversion and crime often go hand in hand. The criminal, released after long confinement, often rapes the aged or commits acts of bestiality.

As a matter of fact, however, the translator of this book is entitled to credit for having done his work faithfully and well. He is an expert in diseases of the nervous system and as a consequence is familiar with the class of cases herein treated of. He is absent in Europe at the present time, probably with a view to further enrich his mental storehouse from the granaries of the old world.

---

A Manual of the Modern Theory and Technique of Surgical Asepsis. By Carl Beck, M. D., Visiting Surgeon to St. Mark's Hospital and to the German Poliklinik of New York City, etc. Small 8vo, pp. 300. With sixty-five illustrations in the text and twelve full-page plates. Price, $1.25 net. Philadelphia: W. B. Saunders, 925 Walnut street. 1895.

Bacteriology has so completely revolutionized the details of the practice of surgery that it becomes necessary to issue separate treatises dealing with the technique of surgical cleanliness. This manual is commended as one of the best of its kind; indeed, it is almost unique, in that it deals with asepsis as contradistinguished from antisepsis. It is, however, true that it is not possible to consider asepsis independently of antisepsis.

The directions that Beck gives for carrying out an ideal surgical asepsis are, in the main, to be commended; and, though it be difficult to adhere to these in every case and under every condition, yet it must be admitted that the ideal must be obtained as nearly as environment will permit. Here comes in the importance of first care to the injured. Much harm is done to wounds by surgical neglect or improper surgical care at first hands. To this end it would be advisable in cities like Buffalo, where the conditions are such as to justify it, to employ an electric railway ambulance in conveying the wounded from the scene of accident or the railway station to the hospital. In case this is done the injured person can receive on board the ambulance adequate aseptic surgical care at first hands. The trolley ambulance is, to all intents and purposes, a temporary hospital supplied with surgeons, nurses and attendants and is equipped with dressings, sterilized water and everything needed for comfort, safety and success.

We think Beck has given too much prominence to iodoform as an antiseptic drug. Its odor is objectionable, it is sometimes poisonous and its place can be supplied by other agents that are quite as good, if not better.

We commend this book to the young practiser of medicine and surgery as a safe guide.

---

MEDICAL GYNECOLOGY. A Treatise on the Diseases of Women from the Standpoint of the Physician. By ALEXANDER J. C. SKENE, M. D., Professor of Gynecology in the Long Island College Hospital, Brooklyn, N. Y.; formerly Professor of Gynecology in the New York Post-Graduate Medical School; Gynecologist to the Long Island College Hospital, etc., etc. Octavo, pp. vi.—529. With illustrations. New York: D. Appleton & Co. 1895.

In examining this book one is at a loss to determine just why it has been written. It must be admitted that there is much of interest and instruction between its covers, but what there is in so-called medical gynecology to demand a separate volume of over 500 standard octavo pages it is beyond our ken to discover. The fact is, there is too much gynecological literature of a text-book character put upon the market. However, since the book is before us, we can give the reader an outline of its scope by stating that the volume is arranged in three parts. Part I. deals with the primary differentiation of sex, development and growth during early life, the conditions favorable to the evolution of normal organization and the attainment of a healthful puberty. This involves the discussion of heredity and environment, including care in childhood, mental and physical education and culture, together with necessary care during the transition from girlhood to womanhood.

Part II. treats of the characteristics of sex, the adaptation of structure to function, the predisposition to particular diseases, and the causes of certain affections peculiar to women. Then follow all the functional and organic diseases common to the period of active functional life of women, that naturally fall under the observation and care of the physician.

Part III. discusses the menopause, or the transition from middle life to old age, and the diseases of that period. There is much philosophizing in the first part, and there is much relating to sexual ethics in the second part, while there is in all three parts a display of learning and a talent for teaching which has made the author justly famous.

This is a book that will be appreciated more especially by teachers, and it is presumed that all such will hasten to avail themselves of the opportunity which the publishers have given them to enrich their libraries.

---

Eighth Annual Report of the Managers of the St. Lawrence State Hospital for the Year 1894. Albany: James B. Lyon, State Printer. 1897.

This report comprises an octavo volume of 210 pages. One half of the book is devoted to the business interests of the institution, including a special report of the state commissioner in lunacy. From this latter we learn that the salary of the medical superintendent is $5,000, and the salaries of the assistant physicians range from $1,800, paid the first, down to $1,200, paid the fourth and the female assistant physicians. We presume the same salaries are paid in the other state hospitals, and the sums are certainly not extravagant, considering the character of the services rendered and the risks incurred.

The last half of the book is devoted to scientific reports of special cases or conditions. Many of these are illustrated, and altogether it is a most satisfactory report. It differs from the majority of such in its scientific value and may serve as a model.

---

The Treatment of Wounds, Ulcers and Abscesses. By W. Watson Cheyne, M. B., F. R. S., F. R. C. S., Professor of Surgery in King's College, London. In one 12mo volume of 207 pages. Cloth, $1.25. Philadelphia: Lea Brothers & Co. 1895.

This small treatise is written by the hand of a master whose knowledge of his work is not surpassed by any living surgeon. He describes in this book the methods which he always employs and which he knows to be efficient and which he believes to be the simplest consistent with certainty and results.

The first one hundred pages are devoted to the consideration of wounds in which detailed directions are given for the aseptic treatment of all injuries—wounds received by accident and wounds made by the surgeon. In speaking of dressings, on page 77, Cheyne says:

I cannot but think that a good deal of the sepsis which occurs in wounds and which is attributed to stitches, catgut, etc., is really in part due to the way in which these wounds are sprinkled with dry iodoform and in which iodoform is trusted as an antiseptic after dressing. As the iodoform has no antiseptic power, it follows that when it is kept in bottles, exposed to dust, etc., it will contain living organisms, and when sprinkled on a wound from such bottles will convey these organisms to the wound and this has actually occurred in many cases.

The next fifty-five pages are devoted to the treatment of ulcers, and the final thirty pages to the treatment of abscesses. This is a valuable contribution to the literature of the subjects with which it deals.

---

INTERNATIONAL CLINICS. A Quarterly of Clinical Lectures on Medicine, Neurology, Surgery, Genito-urinary Surgery, Gynecology, Pharyngology, Rhinology, Ophthalmology, Laryngology, Obstetrics, Otology and Dermatology. By professors and lecturers in the leading medical colleges of the United States, Great Britain and Canada. Edited by JUDSON DALAND, M. D. (Univ. of Penna.), Philadelphia, Instructor in Clinical Medicine and Lecturer on Physical Diagnosis in the University of Pennsylvania; Assistant Physician to the University Hospital; Physician to the Philadelphia Hospital and Fellow College of Physicians, Philadelphia. J. MITCHELL BRUCE, M. D., F. R. C. P., London, England, Physician to, and Lecturer on, Therapeutics at the Charing Cross Hospital. DAVID W. FINLAY, M. D., F. R. C. P., Aberdeen, Scotland, Professor of Practice of Medicine in the University of Aberdeen; Physician to, and Lecturer on, Clinical Medicine in the Aberdeen Royal Infirmary; Consulting Physician to the Royal Hospital for Diseases of the Chest, London. Volume I. Fifth series. 1895. Royal 8vo, pp. xii.—354. Philadelphia: J. B. Lippincott Co. 1895.

In this volume Dr. Charles Cary, of Buffalo, contributes a lecture on parenchymatous nephritis and diabetes, and Dr. M. D. Mann lectures on menorrhagia due to chronic endometritis and amenorrhea. These comprise the local contributions to this number. Other lectures of importance are, one on irritable bladder, by Clinton Cushion, of San Francisco; the chemistry of digestion, by Winslow Anderson, of London, and the treatment of lateral curvature of the spine, by Edward H. Bradford, of Boston. The latter is profusely illustrated by twenty-two figures. This volume is a fitting complement to those that have preceded it.

---

REPORT OF THE COMMISSIONER OF EDUCATION FOR THE YEAR 1892–3. In two volumes. Washington: Government Printing Office. 1894.

From this report of the commissioner of education we learn that the total number of pupils in schools of all grades, public and private, during the school year which it covers, was 14,714,933 or

22.69 per cent. of the population. This number, however, does not include the pupils enrolled in evening schools, nor in schools of art or industry, in business colleges, in schools for defective classes nor in the Indian schools. These make an aggregate of more than 300,000 pupils and increase the total in schools to over 15,000,000 pupils. Teachers and educators cannot fail to be interested in this report which embraces every feature of education and contains valuable statistics on all of its subdivisions.

---

Twenty-Eighth Annual Report of the State Board of Charities for the Year 1894. Transmitted to the Legislature March 25, 1895. Albany: James B. Lyon, State Printer. 1895.

The annual report of this body always possesses interest to those connected with eleemosynary institutions or those who are in sympathy with their conduct or prosperity. Moreover, the work of this board stands in intimate relationship to state economics; hence it deserves to be fostered by the governing powers, executive and legislative.

This volume contains the report and recommendations of the special committee appointed to investigate the management of the State Reformatory at Elmira. In view of the great interest that is just now taking in this matter, we have no doubt this report will be very widely read.

---

Manual of General Medicinal Technology, including Prescription Writing. By Edward Curtis, A. M., M. D., Emeritus Professor of Materia Medica and Therapeutics, College of Physicians and Surgeons, Medical Department of Columbia College, in the City of New York. Third edition, conforming to the U. S. Pharmacopeia of 1894. Pocket size (Wood's Pocket Manual Series), 245 pages. Price, $1.00.

The little manual above described, having reached a third edition within six years from its first appearance, may justly be classed as a popular reference book. Any attempt to improve the topics belonging to medicinal technology or prescription writing is to be commended. This author has contributed much to that end on these subjects. This edition is made to conform to the U. S. Pharmacopeia of 1890. The size of the book is convenient for the pocket.

---

Transactions of the Sixteenth Annual Meeting of the American Laryngological Association, held in the City of Washington, D. C., May 30, 31 and June 1, 1894. Price, $2.50. New York: D. Appleton & Co. 1895.

This volume contains the papers read and discussions had thereon at the sixteenth annual meeting of the American Laryngological Association. They have been published in the *New York Medical Journal*, hence are familiar to the medical profession.

The work done by this association is always of a high order and the year in question furnishes no exception to the rule. Many of the papers published are well illustrated. The book should be in the hands of every physician who practises laryngology.

---

IMMUNITY, PROTECTIVE INOCULATIONS IN INFECTIOUS DISEASES, AND SERUM-THERAPY. By GEORGE M. STERNBERG, M. D., LL. D., Surgeon-General U.S. Army, Ex-President American Public Health Association, Honorary Member of the Epidemiological Society of London, of the Royal Academy of Medicine at Rome, of the Academy of Medicine at Rio Janeiro, of the Société d'Hygiene, etc., etc. One volume, 325 pages, 8vo, bound in extra muslin, beveled edges, uniform, with the other volumes of our *Medical Practitioners' Library*. Price, $2.50 ; in flexible morocco, $3.25. New York : William Wood & Co. 1895.

No one is more competent to treat of the subjects considered in this book than Surgeon-General Sternberg. He is a practical bacteriologist of the first order as well as a scientific man in training, method and habit.

Sternberg divides his work into two parts. In Part I. susceptibility and immunity are considered. Natural immunity is an interesting question, and is very plainly stated in the opening chapter of this book. Acquired immunity is another branch of the subject that is ably treated here. An extended bibliographical list is added at the close of this part.

Protective inoculations in infectious diseases and serum-therapy are considered in Part II., which is the main portion of the book. In separate chapters, Sternberg considers the several diseases in man and the lower animals in which these inoculations apply. At the present time a growing interest is everywhere obtaining in relation to serum-therapy, especially in diphtheria and cholera, as well as a few other diseases ; hence this book becomes of special interest to the general profession of medicine and is likely to be widely read.

---

URINALYSIS, Including Blanks for Recording the Analysis and Microscopic Examination of the Urine. For Medical Practitioners, Life Insurance Examiners and Specialists. Arranged by JOSEPH C. GUERNSEY, A. M., M. D. Philadelphia : J. B. Lippincott Co. 1895.

This book will tend to promote accuracy in urinalysis, the importance of which in diagnosis as well as prognosis and treatment is not easy to estimate. The volume gives a list of apparatus and chemical reagents ; then follows chemical tests in urinalysis ; next the centrifuge in urinalysis, and finally diet. Then follow the record blanks, to the number of 500, which, however, are preceded by an alphabetical index in which to enter the patient's name and page.

The book is unique and will be found of great benefit to both general practitioner and specialist.

A Book of Detachable Diet Lists and a Sick-room Dietary. Compiled by Jerome B. Thomas, A. B., M. D. Price, $1.50. Published by W. B. Saunders, 925 Walnut street, Philadelphia. 1895.

The dietary lists in this book are arranged with reference to (1) albuminuria, (2) anemia and debility, (3) constipation, (4) diabetes, (5) diarrhea, (6) dyspepsia, (7) fevers, (8) gout or uric acid diathesis, (9) obesity, (10) tuberculosis. At the end are a number of leaves devoted to sick-room dietary, that give instructions for the preparation of many articles of food which can be detached and handed to the person in charge. These blanks are an aid to the busy practitioner of medicine ; they enable him to economize his time and tend to insure accuracy in carrying out his directions.

---

Index of Medicine. By Seymour Taylor, M. D., Member Royal College of Physicians ; Senior Assistant Physician to the West London Hospital. In one large 12mo volume of 801 pages, with 35 engravings. Cloth, $3.75. Philadelphia : Lea Brothers & Co.

The author states that his object in compiling this work was to create a handy manual for students in preparing for their final examinations in medicine at the various examining boards. It cannot, therefore, be considered as a text-book, but the author hopes that students may find it a useful supplement to the various treatises on medicine. It is not so much an index as it is a concise volume on the practice of medicine. The author has succeeded in producing just what he intended to create and it merits a wide popularity on the lines marked out.

---

Transactions of the Antiseptic Club. Reported by Albert Abrams, a member of the San Francisco Medical Profession. Illustrated. Price, $1.75. New York : E. B. Treat, 5 Cooper Union. San Francisco : Johnson & Emigh. 1895.

This book seems to have been written as a bit of sarcasm on the germ theory of disease. It is a volume that Mr. Tait would enjoy in the extreme and we would advise the author to send him a complimentary copy. Viewed from any standpoint it contains sufficient mirth-provoking humor to prove entertaining to the most enthusiastic bacteriologist as well as the disbeliever in germs as propagators of disease. Let everybody purchase it and enjoy a good laugh.

---

Transactions of the Medical Society of the State of North Carolina. Forty-first annual meeting, held at Greensboro, N. C., May 15, 16, 17, 1894. Wilmington, N. C.: Jackson & Bell. 1894.

The transactions of the medical society of the old North state is now, as always, replete with interest. Besides the usual professional papers it contains the report of the state board of health and also the minutes of the meeting of the state board of medical examiners. It, however, wastes time and space in printing the code of medical ethics. If the money expended in this way had been devoted to binding the book in cloth it would have contributed to a more permanent preservation of a valuable volume.

---

La Pratique des Maladies du Cœur et de L'Appareil Circulatoire dans les Hopitaux de Paris. Aide-Mémoire et Formulaire de Thérapeutique Appliquée par Le Professeur Paul Lefert. Paris: Librairie J. B. Bailliére et Fils, Rue Hautefeuille, 19 près le Boulevard Saint-Germain. 1895.

The series of manuals of practical medicine issued by MM. Bailliére et Fils consists of twelve volumes. The price of each volume is three francs. This number deals with diseases of the heart and the circulatory apparatus and gives a résumé of treatment as practised in the hospitals of Paris. It is a valuable little book to possess and is of a size convenient to carry in the pocket.

---

Twentieth Annual Report of the Secretary of the State Board of Health of the State of Michigan for the fiscal year ending June 30, 1892. Lansing: Robert Smith & Co., State Printers and Binders. 1894.

The Michigan state board of health is one of the most efficient in the United States, and especially has its secretary attained renown as a sanitarian and as an able executive officer. The scientific character of this report is of a high order. It is a great pity that politics in New York so enter into the question that an efficient secretary, like Dr. Baker, or as Dr. Lewis Balch proved himself, cannot be retained indefinitely.

---

Weekly Abstract of Sanitary Reports issued by the Supervising Surgeon-General M. H. S. Volume IX., Numbers 1 to 52. Washington: Government Printing Office. 1895.

This volume comprises the valuable sanitary reports issued by the supervising surgeon-general of the marine hospital service every week during 1894. It is a matter of great convenience to have them bound together in this manner. A voluminous index makes the subjects of easy reference.

All sanitarians and public health officers will be greatly interested in possessing this book.

---

### BOOKS RECEIVED.

A Guide to the Aseptic Treatment of Wounds. By Dr. C. Schimmelbusch, Assistant in the Royal Surgical Clinic of the University of Berlin. Preface by Prof. E. Von Bergmann. Translated from the second revised German edition, with express permission of the author, by Frank J. Thornbury, M. D., Lecturer on Bacteriology, University of Buffalo, N. Y., etc. Octavo, pp. xii.—233, with forty-three illustrations. New York: G. P. Putnam's Sons, 27 West Twenty-third street. 1895.

Transactions of the American Association of Obstetricians and Gynecologists. Volume VII., for the year 1894. Octavo, pp. 530. Price, cloth, $5.00 ; half-Russia, $6.00. Philadelphia : Wm. J. Dornan, Printer. 1895.

The Care of the Baby. A Manual for Mothers and Nurses, containing practical directions for the management of infancy and childhood in health and disease. By J. P. Crozer Griffith, M. D., Clinical Professor of Diseases of Children in the Hospital of the University of Pennsylvania ; Professor of Clinical Medicine in the Philadelphia Polyclinic and School for Graduates in Medicine ; Physician to the Children's Hospital, etc. Small 8vo, pp. 392. Price, $1.50. Philadelphia : W. B. Saunders, 925 Walnut street. 1895.

Transactions of the Medical Association of Central New York, Twenty-seventh Annual Meeting, Buffalo, N. Y., October 16, 1894. BUFFALO MEDICAL JOURNAL Print. 1895.

The Deformities of the Human Foot, with Their Treatment. By W. J. Walsham, M. B., C. M. Aberd., F. R. C. S., Eng., Senior Assistant Surgeon, Surgeon in Charge of the Orthopedic Department and Lecturer in Anatomy St. Bartholomew's Hospital, etc., etc., and William Kent Hughes, M. B., Lond., M. B. Melb., M. R. C. S., Eng., L. R. C. P. Lond., Orthopedic Surgeon St. Vincent's Hospital ; Assistant Surgeon Children's Hospital, Melbourne ; Surgical Tutor Trinity College, Melbourne, etc., etc. Extra muslin 8vo, $4.50. New York : William Wood & Company. 1895.

